# Impact of duration of cyclic heat stress exposure at different ages on growth performance, recovery, and histopathology in broilers

**DOI:** 10.1016/j.psj.2026.107367

**Published:** 2026-07-02

**Authors:** A. Papanikolaou, E.M.J.M. Kampschoer, M.G.R. Matthijs, S. Khempaka, E.A.W.S. Weerts, J.T. Schonewille

**Affiliations:** a Department of Population Health Sciences, Faculty of Veterinary Medicine, Utrecht University, Yalelaan 7, 3584 CM Utrecht, the Netherlands; b School of Animal Technology and Innovation, Institute of Agricultural Technology, Suranaree University of Technology, Nakhon Ratchasima 30000, Thailand; c Department of Biomolecular Health Science, Faculty of Veterinary Medicine, Utrecht University, Yalelaan 1, 3584 CM Utrecht, the Netherlands

**Keywords:** Broiler, Cyclic heat stress, Recovery, Mortality, Cardiorespiratory histopathology

## Abstract

Heat stress (HS) poses a significant challenge to broiler welfare and growth. While the effects of constant (24 hr/day) HS are well documented, the consequences of HS duration at specific ages and the capacity for subsequent recovery, remain less understood. Therefore, 630 one-day-old Cobb500 broilers were assigned to 7 treatments and reared during a 6 week period to investigate the effects of varying HS duration on broiler growth, and recovery at different ages of exposure. Broilers were housed either in a climate-controlled (CC) barn or a natural cyclic HS open-sided (OS) barn. Treatments included broilers continuously housed for 6 weeks in CC (6CC) or OS (6OS). In addition, three other treatments were transferred from CC to OS for the final 3 weeks (3OS), 2 weeks (2OS), or 1 week (1OS) of the experiment. The last two recovery treatments (REC1, REC2) were placed in the CC house followed by 1 or 2 weeks in the OS house, from day 21 or 28 until 35, before returning to CC for the final week of the experiment. Each treatment included six replicates, with 15 broilers per replicate pen at the start of the experiment. Cloacal temperature, growth performance, mortality, plasma corticosterone, protein carbonyls, and histological morphology of the heart and lung were evaluated. Exposure to HS increased the cloacal temperature by at least 1.5°C at all ages (*P* < 0.001). Sudden exposure to HS at 36 days of age (1OS) resulted in severe mortality (67%) compared to control (6CC) and broilers exposed to HS at younger age (3OS and 2OS). HS consistently reduced body weight (BW) and average daily feed intake (ADFI); 6OS broilers finished 40.1% lighter than 6CC broilers (*P* < 0.001). Upon returning to thermoneutral conditions, REC1 and REC2 broilers immediately resumed growth rates comparable to controls but did not exhibit compensatory growth to recover the accumulated BW deficit. Very short-term HS exposure (1OS) triggered a spike in plasma corticosterone (25.42 nmol/L) and protein carbonyls (1.92 nmol/mg) compared to 6CC controls (*P* < 0.001). Descriptive histological examination revealed that subendocardial erythrocyte accumulations were increased in 6OS hearts and were absent in CC-housed broilers. Taken together, sudden HS exposure at 36 days of age poses an unmanageable threat to survival. While broilers recover from HS quickly, lost performance is not regained through compensatory growth within the 6 week period.

## Introduction

In light of global climate change, the issue of heat stress (**HS**) in broilers is important. Not only from a growth performance (Liu et al., 2020) and thus economic perspective (St-Pierre et al., 2003), but also from an animal welfare perspective (Panel et al., 2023). Currently, most HS studies use constant heat challenge models, although their practical relevance can be questioned (de Souza et al., 2016). In commercial settings, HS typically occurs for only part of the day and therefore a cyclic HS model was applied in the present study. Beside the duration of HS during the day, the age during exposure is critical.

To date, there is a dearth of studies on HS applied during specific periods within the growth cycle. Interpretation of results across independent studies is considered a challenge due to the wide range of factors that can influence the impact of HS on production and welfare, such as genotype, relative humidity and ambient temperature, age at exposure, exposure time during the day, and especially the large variation in length of the experimental period studied (Renaudeau et al., 2012). Moreover, the broilers’ capacity to survive, grow or even compensate for the reduced growth after a period of HS exposure remains insufficiently understood (Jinxue et al., 2023). For the purposes of this study, the time period following HS exposure will be referred to as recovery throughout the paper.

In addition, animal welfare is a key consideration in modern livestock production. Although the use of plasma corticosterone concentrations to assess animal welfare can be disputed (Kannan and Mench, 1996), the inverse relationship between the plasma corticosterone concentration and animal welfare is generally accepted (Blas and Fairhurst, 2022). Nevertheless, it should be kept in mind that plasma corticosterone concentration is related to stress which is clearly not equivalent to welfare. It is known that broilers respond to HS by increasing plasma corticosterone concentrations (Beckford et al., 2020), but the influence of HS timing and duration on corticosterone responses under cyclic conditions remains unclear. Thus, it is considered opportune to include the plasma corticosterone concentration in the present study. In addition, HS is known to induce oxidative stress (Azad et al., 2010) and therefore plasma protein carbonyl products were measured as an indicator of oxidative stress.

Beside the oxidative stress and corticosterone response, one of the underlying mechanisms to explain a HS induced reduction in growth performance involves the digestibility of the dietary macro nutrients. Larbier et al. (1993) and Bonnet et al. (1997), for instance, reported on a depressed nutrient digestibility when broilers were exposed to constant HS. The effect of specifically cyclic HS and duration on nutrient digestibility remains unclear, therefore, digestibility was measured during the final week of the current study.

Furthermore, a heat challenge can potentially negatively affect the tissues of the cardiorespiratory system of the broilers. Broilers respond by panting and gular fluttering to enhance heat loss (Papanikolaou et al., 2025) and increase cardiac output (Nawaz et al., 2021). HS-induced histological changes of heart and lungs are poorly described in the literature and still a known knowledge gap in the pathology of HS.

Taken together, the objective of this study was to evaluate the effects of the timing and duration of cyclic HS on growth performance and recovery, together with associated physiological, metabolic and intestinal responses. We hypothesize that cyclic HS would reduce growth performance and impair recovery, and that these effects would be accompanied by increased corticosterone concentrations, elevated oxidative stress, a decreased nutrient digestibility, and changes in organ morphology of the lung and heart of broilers.

## Material and methods

### Ethical considerations

The current experiment was approved by the Animal Ethics Committee of Suranaree University of Technology Isan (SUT, Approval number: SUT-IACUC-008/2023) and based on the Ethics of Animal Experimentation of the National Research Council of Thailand.

The broilers were monitored four times a day between 8:00 and 21:00 hr. The broilers’ behaviour was assessed to check if any humane endpoints, as described by Beckford et al. (2020), were present: severe panting or no panting despite HS, or if they were non-responsive to human approach or touch, and were glassy-eyed and stupefied, or were found dead. Mortality was recorded as the sum of broilers that either were found dead during the experiment, or reached the humane endpoint and were removed from the experiment. Broilers removed for sampling were not classified as mortality.

### Animals, pens and diets

The experiment was conducted in Thailand (approx. 14°52′N, 102°00′E) during March and April. A total of 630 one-day-old Cobb500 broilers were obtained from a commercial hatchery (Chonburi, Thailand). The hatchlings were acquired as males, based on feather-sexing. All broilers were vaccinated at the hatchery against Marek’s disease (FATRO S.p.A., Bologna, Italy) and 7 days after hatch, they were vaccinated against Newcastle disease and infectious bronchitis (FATRO S.p.A., Bologna, Italy). At day 14 after hatch, the broilers were vaccinated against infectious bursal disease (FATRO S.p.A., Bologna, Italy).

All broilers were housed in pens (1.12 m × 0.82 m) on concrete floors covered with rice husks (7.4 kg/m2), previously disinfected with a solution containing 15% glutaraldehyde and 10% ammonium chloride. Throughout the experiment the stocking density never exceeded 42 kg broiler/m^2^ (EU/2007/43/EC).

The ingredient- and nutrient composition of the diets (Table 1) was identical across the treatments. Consisted of a starter diet (day 1 to 12 of age), a grower diet (day 13 to 28 of age) and finisher diet (day 29 to 43 of age). All diets were provided in mash form and were formulated to meet, or exceed, the nutrient requirements as recommended by the Cobb-Vantress (2021) Cobb Broiler Management guide. During the first 10 days after hatch, each pen was equipped with tray feeders and from day 11 onwards, a round-bottomed (⌀ 42 cm) hanging feeder was used (one per pen). Throughout the experiment, water was provided using bell drinkers (one per pen, ⌀ 30 cm). Feed and water were available for *ad libitum* consumption at all times and were replenished twice daily at 08:00 and 16:00 hr.

**Table 1:** Ingredient and calculated nutrient composition of the diets fed during day 1 to 12 (starter), day 13 to 28 (grower) and day 29 to 42 (finisher). Unless indicated otherwise, all values are expressed as % as fed.

	Diets			
	Starter	Grower	Finisher 1	Finisher 2^1^
Ingredients				
Corn	56.48	63.13	64.43	64.43
Soybean meal (44% CP)	36.20	31.00	28.85	28.55
Palm oil	1.80	1.12	2.41	2.41
Calcium carbonate	1.23	1.23	1.15	1.15
Monocalcium phosphate	2.10	1.27	1.15	1.15
NaCl	0.52	0.52	0.52	0.52
Premix^2^	0.50	0.50	0.50	0.50
Titanium dioxide	-	-	-	0.30
L-lysine HCl 79%	0.34	0.35	0.29	0.29
DL-methionine	0.39	0.37	0.32	0.32
L-threonine	0.21	0.18	0.13	0.13
L-Arginine	0.14	0.16	0.13	0.13
L-isoleucine	0.04	0.06	0.03	0.03
L-valine	0.05	0.11	0.09	0.09
Calculated composition				
ME (kcal/kg)	2900	2950	3050	3050
Crude protein	21.51	19.75	18.66	18.66
Crude fat	3.96	3.47	4.78	4.78
Crude fibre	3.82	3.60	3.48	3.48
Calcium	0.96	0.80	0.74	0.74
Phosphorus	0.83	0.64	0.61	0.61
Available phosphorus	0.58	0.40	0.37	0.37
Digestible Lys	1.26	1.16	1.06	1.06
Digestible Met	0.67	0.63	0.57	0.57
Digestible Met + Cys	0.94	0.88	0.82	0.82
Digestible Thr	0.86	0.78	0.70	0.70
Digestible Arg	1.36	1.25	1.16	1.16
Digestible Ileu	0.81	0.75	0.69	0.69
Digestible Val	0.89	0.88	0.82	0.82
Digestible Tryp	0.26	0.23	0.22	0.22
Digestible His	0.49	0.45	0.43	0.43
Digestible Leu	1.59	1.49	1.44	1.44

1 Except for the replacement of soybean meal by TiO_2_ (0.3% as fed) as marker for digestibility, the ingredient compositions of finisher1 and finisher 2 were identical.

2 Premix provided the following (per kg of diet): vitamin A (trans-retinyl acetate) 15000 IU; vitamin D3 (cholcalciferol) 3000 IU; vitamin E (DL-a-tocopherol) 25 IU; vitamin K3 (menadione nicotinamide bisulphite) 5 mg; thiamine (thiamine-mononitrate) 2 mg; riboflavin 7 mg; pyridoxine (pyridoxine. HCl) 4 mg; vitamin B12 (cyanocobalamin) 25 µg; pantothenic acid (D-Ca pantothenate) 11.04 mg; niacin (nicotinic acid) 35 mg; folic acid 1 mg; biotin 15 µg; choline chloride 250 mg; Cu (copper sulfate) 6.4 mg; Mn (manganese oxide) 100 mg; Zn (zinc oxide) 75 mg; Fe (iron sulfate) 0.40 g; I (calcium iodate) 0.40 mg; Se (sodium selenite) 0.361 mg.

### Experimental design

Upon arrival, all hatchlings were weighed and stratified by body weight (**BW**) to achieve similar initial mean BW across all pens. Then, six individual pens (15 broilers/pen), were randomly assigned to one of the seven treatments. Directly thereafter, each individual broiler was tagged and weighed again. The experimental broilers were either housed in an open-sided (**OS)** barn or a climate-controlled (**CC**) barn. The broilers housed in the OS barn were exposed to the prevailing climatological conditions, characterized by natural high temperatures during the day. No additional heat was provided in the OS barn, except for the first week after hatch, i.e., the brooding period. In contrast, the temperature in the CC barn was regulated using three air-conditioning units (York air conditioner, 40000 btu, York, USA). The temperature was adjusted daily following the guidelines recommended by Cobb-Vantress (Cobb Broiler Management Guide, 2021). Furthermore, an industrial dehumidifier (YDA-890EB, Parkoo, China) was used to avoid relative humidity ≥ 70%. Brooding heat was provided to all the broilers during the first week after hatch. In the CC barn, a gas heater was used to ensure minimum brooding temperatures as recommended by Cobb-Vantress (Cobb Broiler Management Guide, 2021). In the OS barn one infrared heat lamp bulb (175 W) per pen was placed ∼20 cm above the broilers.

Fluorescent lighting, utilizing high-frequency bulbs to prevent flicker-induced stress, was provided throughout the experiment. The lighting program consisted of 1 h of darkness during the brooding period (days 1–7), followed by 8 h of darkness (days 8–20), 7 h of darkness (days 21–35), and 6 h of darkness (days 36–42). In the OS barn supplemental artificial light was provided before sunrise and after sunset to ensure the photoperiod remained identical to the CC barn.

The experiment lasted 6 weeks and broilers were allocated to seven experimental treatments. Broilers subjected to the first two treatments were housed either continuously in the CC barn (**6CC**) or the OS barn (**6OS**) (Table 2). Broilers subjected to the following three treatments were moved from the CC barn to the OS barn for the final 3 weeks, 2 weeks or 1 week of the experiment (designated **3OS, 2OS**, and **1OS**, respectively). The final two treatments were recovery treatments. These treatments underwent sequential transitions from the CC barn to the OS barn, and ultimately back to the CC barn. In the **REC1** treatment broilers were subjected to 4 weeks CC, 1 week OS, and 1 week CC, and in the **REC2**, broilers were subjected to 3 weeks CC, 2 weeks OS, and 1 week CC. All translocations from CC to OS, or *vice versa*, took place between 7:30 and 10:30 hr (after the weighing of the animals) on the first day of the experimental week in question (i.e., days 22, 29 and 36).

**Table 2:** Overview of the experimental treatments. The broilers were either housed in a climate-controlled barn (CC) or in an open-sided barn (OS) where broilers were subjected to the prevailing environmental climate. The values represent the mean, 24 h temperature (°C, ± standard deviation) during the week in question.

		Experimental treatments^1^						
Week	Cobb500 guideline^2^	6CC	6OS	3OS	2OS	1OS	REC1	REC2
1	30.1	CC: 32.3 ± 2.7	OS: 32.7 ± 3.7	CC: 32.6 ± 2.6	CC: 32.7 ± 2.8	CC: 32.3 ± 2.6	CC: 32.4 ± 2.7	CC: 32.5 ± 2.7
2	27.9	CC: 27.1 ± 0.9	OS: 31.1 ± 4.8	CC: 27.0 ± 1.0	CC: 25.5 ± 1.2	CC: 26.5 ± 1.0	CC: 26.9 ± 0.9	CC: 27.1 ± 0.9
3	25.7	CC: 26.2 ± 1.2	OS: 31.5 ± 4.5	CC: 25.5 ± 1.2	CC: 25.8 ± 1.4	CC: 24.8 ± 1.4	CC: 26.5 ± 1.3	CC: 26.0 ± 1.2
4	23.5	CC: 23.9 ± 2.1	OS: 31.9 ± 3.9	OS: 31.3 ± 4.6	CC: 23.4 ± 2.6	CC: 22.8 ± 2.1	CC: 23.7 ± 2.2	OS: 31.2 ± 4.3
5	21.7	CC: 22.3 ± 2.5	OS: 32.8 ± 3.9	OS: 32.5 ± 4.1	OS: 31.8 ± 4.9	CC: 22.4 ± 4.6	OS: 32.0 ± 4.8	OS: 32.2 ± 4.2
6	20.0	CC: 21.6 ± 1.9	OS: 31.0 ± 4.1	OS: 30.6 ± 4.3	OS: 30.8 ± 4.1	OS: 29.9 ± 5.0	CC: 22.0 ± 2.8	CC: 21.6 ± 2.6

1 6CC and 6OS = 6 weeks housing of the broilers in the CC or OS barn, respectively. 3OS, 2OS and 1 OS = broilers that were housed in the OS barn for 3, 2 or 1 weeks, respectively, and for the previous 3, 4 or 5 weeks in the CC barn, respectively. REC1 = 4 weeks housing of the broilers in the CC barn, followed by 1 week housing in the OS barn, followed by 1 week in the CC barn. REC2 = 3 weeks housing of the broilers in the CC barn, followed by 2 weeks housing in the OS barn, followed by 1 weeks in the CC barn ^2^ The temperature recommendations are according to the Cobb500 management guide and were adapted based on the stocking density of the pens.

Throughout the experiment the temperature and relative humidity were monitored and recorded automatically at 10-minute intervals using data loggers (Tempmate® S2, Heilbronn, Germany). Within each treatment, two data loggers were attached to the pen mesh at ∼ 25 cm above the litter. The specific treatment temperature and humidity averages are detailed in Table 2. The temperature in the CC barn decreased gradually from 34°C to 23°C during the first 3 weeks in order to mimic Cobb500 recommendations. Subsequently, the temperature was maintained at 22-23°C. In contrast, the temperature in the OS barn had an average of 31°C throughout the experimental period (Table 2). The temperature readings of all individual treatments are shown in Table 2. An example of the mean daily temperature fluctuation during week 4 is shown in Fig. 1.

**Fig. 1:**
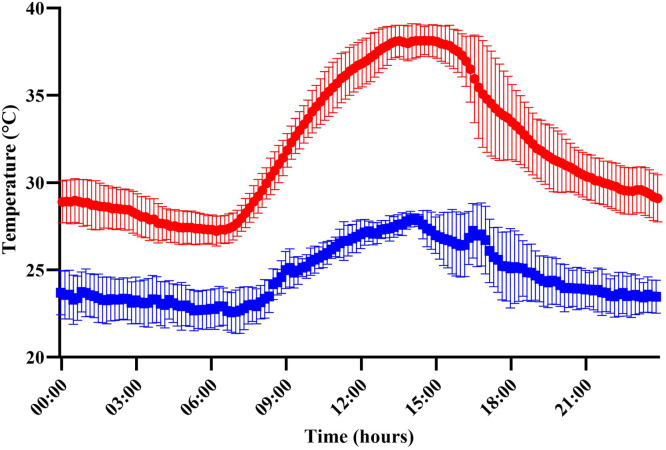
Diurnal pattern of temperature during week 4 in the climate-controlled barn (blue line, CC) and open-sided barn (red line, OS). Each datapoint represents the mean temperature of each hour during week 4. The error bars depict the SD for every hour.

Relative humidity in the CC barn was between 55% to 70%, whereas in the OS barn the relative humidity varied from 40% to 80%. The average relative humidity for the both the CC and OS barn was 62%, although the standard deviation was ±5% and ±13%, respectively. The continuous temperature and relative humidity readings are shown in supplementary Fig. 1.

### Data and sample collection

To minimize cumulative handling stress, interventions were concentrated on the same day whenever possible. The routine individual weighing of all broilers was combined with the translocation events. During these events (between 07:30 and 10:30 hr), all broilers, including those designated to remain in their respective barns, were individually caught, weighed in the vicinity of their pen, and placed into transport cages before being transported to their new barn or returned to their original pen. Furthermore, additional handling required for cloacal temperature measurements and blood collection was strictly limited to one pre-selected broiler per pen to avoid subjecting the entire flock to repeated sampling stress.

On days 8, 15, 22, 29, 36, and 43, between 07:30 and 10:30 hr, all broilers were individually weighed. Individual body weight data were used to select the average birds for sampling. Immediately prior to the weighing of the broilers, the hanging feeders were removed from each pen and feed left overs were recorded. Feeding was resumed right after the weighing of all the broilers.

Cloacal temperatures were recorded using a digital thermometer (SC12, SCALA Electronic GmbH, Germany) on days 21, 28, 35, and 42 (16:00 hr), and days 22, 29, 36, and 43 (08:00 and 16:00 hr). Measurements were taken from one broiler per pen (selected based on the closest match to the most recent mean pen BW), which was tracked across consecutive days. On days 35 and 36, immediately following the 16:00 h measurement, ∼3 mL of brachial blood was collected using a 24-gauge syringe (Sterican®, B. Braun, Germany). Blood was transferred to lithium-heparin tubes on ice, centrifuged within 2 h (1200 × g, 15 min), and the plasma stored at -20°C. Sampled broilers were subsequently euthanized via cervical dislocation for further tissue sampling. The left lung and heart were collected from the 6CC, 6OS and 2OS broilers on day 36. Both organs were fixed for 24-48 hr in 4% formalin and thereafter stored in 70% alcohol.

On day 43, one random broiler per pen of the 6CC, 6OS, 3OS and REC2 treatments was euthanized and ileal digesta were collected from Meckel’s diverticulum to approximately 3 cm proximal to the ileo-cecal junction to determine the apparent ileal digestibility (AID) of dry matter, organic matter, ash, and crude protein.

The AID was evaluated in a targeted subset of four treatments to optimize resources. The 6CC and 6OS groups were selected to establish the baseline and maximum chronic exposure boundaries. The 3OS group was included to assess the impact of an active, intermediate thermal challenge. Additionally, the REC2 group (2 weeks of OS followed by 1 week of CC) was selected to evaluate the capacity for digestive recovery following the removal of the heat stressor.

### Chemical analyses

The dry matter content of the finisher diet (Table 1) was determined by drying at 135°C for 3 hr (AOAC, 1990) and the ash content was determined by combustion at 550°C for 3 hr (Thiex et al., 2012). The N content was determined using the Dumas combustion technique (AOAC, 2006) using a nitrogen analyser (Elementar Analysensysteme GmbH, Hanau, Germany). Crude protein was calculated by multiplying the N content by 6.25. Titanium dioxide content in diets and ileal digesta was determined as described by Short et al. (1996).

Plasma corticosterone concentration was determined, in duplicate, using a commercial corticosterone ELISA kit (Labor Diagnostika Nord ELISA kit, cat. no. MS-E-5400). Absorbance was measured with a microplate reader (DTX880) and concentrations were calculated using Anthos Zenyth Multimode Detectors (v.2.0.0.13) and analysed with GraphPad (v.10), respectively.

The protein carbonyl concentration was measured, in duplicate, using a commercial colorimetric assay kit (Elabscience Protein Carbonyl Colorimetric Assay Kit, cat. no. E-BC-K117-S). Absorbance was measured with a microplate reader (FLUOstar Omega, BMG Labtech, Ortenberg, Germany) and values were converted into concentrations using MARS (v.5.02) and analysed with GraphPad (v.10), respectively.

### Histological examination

Heart and left lung were transversely cut into halves to obtain tissue from the apical- or cranial region of the heart and lung, respectively. Subsequently, the samples were embedded in paraffin and slides were cut and slides were stained with haematoxylin and eosin (H&E) according to standard laboratory procedures. The stained tissue slides were examined using a light-microscope to identify differences between the treatment groups. To optimize tissue processing resources, it was decided that histopathological changes were first to be assessed in the 6CC, 2OS, and 6OS treatments on day 36. With this strategy, the extremes of the treatments were evaluated first, operating on the rationale that an absence of pathology at the maximum exposure duration would imply a similar lack of pathology at intermediate HS exposure lengths. The examination of the tissue slides was conducted independently by three veterinarians, including a board-certified pathologist and a senior poultry researcher, all affiliated to the Faculty of Veterinary Science, Utrecht University, the Netherlands. Each slide was marked with a randomly chosen number and the key to the experimental treatments was unknown to the independent veterinarians. Prior to detailed histological evaluation, each examinator blindly and independently scanned through the tissues to pinpoint potential relevant abnormalities and a plenary calibration session was held to decide which criteria would be taken into account. Subsequently, the examiners independently and again blindly evaluated each sample according to the chosen criteria of interest. Following these independent assessments, the reported findings for each sample were established through discussion to reach examiner consensus.

For the lung tissue, histopathological assessment included the evaluation of presence of cellular infiltrates (inflammatory cells and extramedullary hematopoiesis), presence of fibrosis, presence of blood within the parabronchial lumen and presence of cartilaginous or osseous nodules. Cardiac tissues were examined for degenerative changes of cardiomyocytes, inflammatory cell infiltration, fibrosis, vascular changes, extramedullary hematopoiesis and alterations to the lumen-lining endothelium and underlying lamina propria, with specific attention to subendocardial erythrocyte accumulations.

### Calculations and statistical analysis

Weekly mortality was expressed as the number of dead broilers / the number of surviving broilers in the week in question × 100. Broilers that reached humane endpoints were included into the mortality counts but not the broilers intentionally killed for sampling purposes. Body weight was recorded as the average body weight per broiler, per pen. Average daily gain (**ADG**) was calculated by dividing the total weekly gain by 7. The average daily feed intake (**ADFI**) was calculated by total feed intake from each pen divided by the number of broilers in that pen and was corrected for the mortality. The correction was based on time of death and thus the amount of time that the broiler was able to consume feed. The feed conversion ratio (**FCR**) was calculated as kg feed / kg BW gain. Furthermore, the growth ratio was calculated for each experimental week and was calculated as; (final body weight week_i_) / (initial body weight week_i_), where i = the week in question.

The AID of nutrients was calculated as follows (Kim et al., 2012):

$$\text{AID}\left(\%\text{of}\text{intake}\right)=\left[1-\left(Ti_{\text{diet}}/Ti_{\text{ileal}\text{digesta}}\right)\times \left(\text{Nutrien}t_{\text{ileal}\text{digesta}}/\text{Nutrien}t_{\text{diet}}\right)\right]\times 100\%,$$

where Ti and nutrients are the chemically analysed concentrations in both the diet and ileal digesta, with all concentrations expressed as g/kg.

Throughout, pen is considered as statistical unit. Weekly mortality was analyzed independently for each week using a binomial Generalized Linear Model (GLM) in R (version RStudio 2025.05.0+496). For weeks where complete separation occurred due to 0% mortality in a treatment group, models were fitted using Firth’s penalized likelihood. To control for multiple comparisons across the 6-week trial, overall weekly *P*-values were adjusted using the Benjamini-Hochberg False Discovery Rate (FDR). For weeks demonstrating statistical significance, pairwise differences between treatments were evaluated using a Tukey adjustment.

Prior to hypothesis testing, the assumption of a Gaussian distribution was checked using the Shapiro-Wilk test. BW, ADFI, ADG, growth ratio and cloaca temperature were analyzed using Repeated Measures Mixed-Effects Model in GraphPad Prism (version 10.4.1, 627) using Time, Treatment and Time × Treatment as fixed factors and individual pen as random factor. Upon detection of a statistically significant main effect or interaction (*P* ≤ 0.05), post-hoc pairwise comparisons were conducted utilizing Tukey’s multiple comparisons test to identify significant differences between treatments on the variable involved.

Corticosterone and protein carbonyl concentrations were analyzed using a repeated measures ANOVA with Treatment, Day of sampling and Treatment × Day, as fixed effects and pen as random factor. Significant effects or interactions were followed by Sidak's test for within-treatment time comparisons and Tukey's test for within-day treatment differences. Throughout, results are reported as least squares means and statistical significance was set at *P* < 0.05.

## Results

### Cloacal temperature

The interaction between the boilers’ age and treatment affected the mean cloacal temperature of the broilers (*P* < 0.001). The cloacal temperature was found to be at least 1.5°C higher in broilers housed in the OS barn compared to those in the CC barn (Table 3). An exception to the aforementioned results is the cloacal temperature of broilers subjected to the REC1 and REC2 treatments, which was lower (*P* < 0.012) than the 6CC broilers on day 36 (41.6°C), 8 hr after the transfer to the CC barn, i.e., 40.5°C and 40.4°C, respectively. This effect was not found on day 43 (*P* ≥ 0.990), i.e., 7 days after transfer to the CC barn.

**Table 3:** Cloacal temperature (°C) at 16:00 hr, in broilers either housed in a climate-controlled (CC) barn or an open-sided barn (OS).

	Experimental treatments^1^							SEM
	6CC	6OS	3OS	2OS	1OS	REC1	REC2	
Day								
22	41.4^b^	42.9^a^	42.8^a^	41.6^b^	41.3^b^	41.5^b^	43.1^a^	0.185
29	41.1^b^	43.4^a^	43.3^a^	43.1^a^	41.1^b^	43.4^a^	43.4^a^	
36	41.5^b^	43.9^a^	43.3^a^	43.1^a^	43.4^a^	40.5^c^	40.3^c^	
43	41.6^b^	43.4^a^	43.0^a^	43.1^a^	43.1^a^	41.5^b^	41.7^b^	
***P*-values**								
Treatment	<0.001							
Day	<0.001							
Treatment × Day	<0.001							

a-c Means within each row without common superscript are significantly different (*P* < 0.05).

1 6CC and 6OS = 6 weeks housing of the broilers in the CC barn or OS barn, respectively. 3OS, 2OS and 1 OS = broilers that were housed in the OS barn for 3, 2 or 1 weeks, respectively, and for the previous 3, 4 or 5 weeks in the CC barn, respectively. REC1 = 4 weeks housing of the broilers in the CC barn, followed by 1 week housing in the OS barn, followed by 1 week in the CC barn. REC2 = 3 weeks housing of the broilers in the CC barn, followed by 2 weeks housing in the OS barn, followed by 1 weeks in the CC barn.

Cloacal temperature values of the previous afternoon and morning measurements are available in the supplementary Table 1.

### Growth performance

The interaction between treatment and broiler age had an effect on the body weight of the broilers (*P* < 0.001). Broilers that were exposed to HS in the OS barn had a reduced growth which resulted in a 21.6% lower BW when compared to the broilers allocated to the 6CC treatment at 22 days of age (Table 4).

**Table 4:** Growth performance of broilers either housed in a climate-controlled (CC) barn or an open-sided barn (OS).

	Experimental treatments^1^								SEM
	6CC	6OS	3OS	2OS	1OS	REC1		REC2	
Body weight, g/bird^2^									
Day 22	1024^a^	802^b^	1020^a^	989^a^	1018^a^	980^a^		1004^a^	40.45
Day 29	1732^a^	1286^c^	1502^b^	1699^a^	1747^a^	1681^a^		1499^b^	
Day 36	2569^a^	1683^d^	1915^bc^	2077^bc^	2536^a^	2098^b^		1907^c^	
Day 43	3450^a^	2065^e^	2296^d^	2383^cde^	2780^abcde^	2880^b^		2593^c^	
Average daily feed intake, g/bird/d									
Week 3	102.1^a^	75.4^b^	102.8^a^	101.3^a^	103.7^a^	98.7^a^		100.4^a^	3.62
Week 4	155.9^a^	111.4^c^	119.1^bc^	151.9^a^	156.6^a^	150.8^a^		122.7^b^	
Week 5	202.3^a^	126.6^b^	134.6^b^	140.5^b^	198.8^a^	142.8^b^		138.4^b^	
Week 6	229.3^a^	126.0^c^	157.3^bc^	161.0^c^	144.1^c^	198.7^ab^		176.1^c^	
Average daily gain, g/bird/d									
Week 3	71.2^ab^	50.3^c^	70.6^ab^	69.6^ab^	72.2^a^	67.1^b^		71.8^ab^	3.82
Week 4	101.4^a^	69.2^b^	69.4^b^	99.7^a^	104.4^a^	97.2^a^		70.0^b^	
Week 5	117.8^a^	57.4^b^	59.2^b^	54.7^b^	107.7^a^	58.8^b^		62.9^b^	
Week 6	136.4^a^	50.1^b^	52.8^b^	46.0^b^	9.9^ab^	110.8^a^		98.8^a^	
Feed conversion ratio^3^									
Week 3	1.43^abc^	1.50^a^	1.42^bc^	1.46^abc^	1.43^bc^	1.47^ab^		1.42^c^	0.176
Week 4	1.54^b^	1.61^ab^	1.72^a^	1.52^b^	1.50^b^	1.56^ab^		1.76^a^	
Week 5	1.72^c^	2.22^a^	2.30^ab^	2.65^ab^	1.85^bc^	2.45^a^		2.22^ab^	
Week 6	1.84^b^	2.55^a^	3.06^ab^	4.44^ab^	4.13^ab^	1.80^b^		1.79^b^	
Growth ratio^4^									
Week 3	1.95^ab^	1.79^c^	1.99^ab^	1.98^ab^	1.99^ab^	1.92^b^		1.99^a^	0.018
Week 4	1.69^ab^	1.60^b^	1.48^c^	1.72^a^	1.72^a^	1.71^ab^		1.49^c^	
Week 5	1.48^a^	1.32^b^	1.28^bc^	1.24^c^	1.45^a^	1.25^c^		1.29^bc^	
Week 6	1.34^a^	1.24^b^	1.20^b^	1.17^b^	1.20^ab^	1.38^a^		1.36^a^	
***P*-values**	**BW**	**ADFI**	**ADG**	**FCR**	**Growth ratio**				
Treatment	<0.001	<0.001	<0.001	0.742	<0.001				
Week/Day	<0.001	<0.001	<0.001	0.044	<0.001				
Treatment × Week/Day	<0.001	<0.001	<0.001	0.922	<0.001				

a-e Means within each row without common superscript are significantly different (*P* < 0.05).

1 6CC and 6OS = 6 weeks housing of the broilers in the CC barn or OS barn, respectively. 3OS, 2OS and 1 OS = broilers that were housed in the OS barn for 3, 2 or 1 weeks, respectively, and for the previous 3, 4 or 5 weeks in the CC barn, respectively. REC1 = 4 weeks housing of the broilers in the CC barn, followed by 1 week housing in the OS barn, followed by 1 week in the CC barn. REC2 = 3 weeks housing of the broilers in the CC barn, followed by 2 weeks housing in the OS barn, followed by 1 weeks in the CC barn.

2 Initial body weight was similar (pooled SEM = 0.25, *P* >0.999) between treatments and the mean value across all treatments was 43.3 g/broiler

3 Feed conversion ratio, calculated as g of feed / g of body weight gain.

4 Weekly growth ratio = (final body weight week_i_) / (initial body weight week_i_), where i = week number.

After one week of HS exposure, between day 22 and 29 of age, the broilers in the 3OS and REC2 treatment had a reduced BW of 13.3% and 13.5% (*P* < 0.008), respectively, compared to 6CC. A further reduction of BW as observed during the second week of HS exposure, although the effect was increased to 19.2% and 18.3% at 36 days of age.

The broilers subjected to the 6CC treatment had the highest BW at the end of the experiment (*P* < 0.001). When compared to the 6CC broilers, those exposed to HS conditions for 3 weeks (3OS), had 33.5% lower BW (*P* < 0.001). Broilers that returned to the CC barn for the last week after 1 (REC1) or 2 (REC2) weeks in the OS barn, had 16.5% and 24.8% lower BW (*P* < 0.001) than the 6CC broilers. The 6OS broilers had the lowest BW, finishing 40.1% lighter than the 6CC broilers (*P* < 0.001).

The impact of HS on ADG and ADFI throughout the 6-week period mirrored the patterns observed in BW and were similar affected by the interaction of week of age and treatment (*P* < 0.001). Most notably, sudden exposure to OS during this final week (1OS) resulted in a severe 92.7% reduction in ADG (*P* < 0.001). The FCR was only affected by the week of age (*P* < 0.044).

The growth ratio in week 6 was affected by the interaction between week of age and treatment (*P* < 0.001). Broilers allocated to the 3OS, 2OS and 6OS treatments had 10.5%, 12.7% and 7.5% lower growth ratio when compared to the 6CC broilers (*P* < 0.034) (Table 4). However, the growth ratio of the broilers in treatment REC1 and REC2, that returned to CC housing for that week, was similar (*P* ≥ 0.281) to the broilers in the 6CC treatment in week 6.

### Mortality

Mortality of the broilers subjected to the 6CC treatment was 1.9% (Table 5). This contrasts with the 1OS treatment, where the mortality peaked during week 6 with 66.7%. The majority of these broilers died or were removed within 24 hr of transfer to the OS barn on day 36 (Fig. 2). A gradual increase of mortality was recorded during the experimental period in the 6OS, 3OS and 2OS treatments, and increased even more during the last week, to 30.4% (Table 5). In the broilers subjected to REC1 and REC2, mortality rose after their transfer (day 22 and 29, respectively) and remained constant thereafter.

**Table 5:** Weekly mortality broilers either housed in a climate-controlled (CC) barn or an open-sided barn (OS). Data are presented as treatment means of pen mortality ± standard error of the mean (SEM), with n = 6 replicate pens per treatment group. Broilers removed for sampling purposes were not considered as mortality.

	Experimental treatments^1^							P value^2^	Adj, P value
	6CC	6OS	3OS	2OS	1OS	REC1	REC2		
Week									
1 and 2	0.0 ± 0.0	0.0 ± 0.0	0.0 ± 0.0	0.0 ± 0.0	0.0 ± 0.0	0.0 ± 0.0	0.0 ± 0.0	>0.999	>0.999
3	0.0 ± 0.0	9.0 ± 2.4	1.3 ± 1.3	0.0 ± 0.0#	0.0 ± 0.0	0.0 ± 0.0#	1.3 ± 1.3#	0.004	0.004
4	0.0 ± 0.0	1.7 ± 1.7	6.9 ± 2.6	0.0 ± 0.0	0.0 ± 0.0	0.0 ± 0.0#	8.6 ± 0.2	0.001	0.002
5	0.0 ± 0.0#	8.7 ± 5.3	6.5 ± 3.3	18.9 ± 8.3	0.0 ± 0.0	4.5 ± 3.1	14.3 ± 3.6#	<0.001	<0.001
6	1.9 ± 1.9#^c^	30.4 ± 11.1^b^	30.6 ± 10.3^b^	51.0 ± 13.1^ab^	66.7 ± 15.8^a^	1.7 ± 1.7^c^	0.0 ± 0.0#^bc^	<0.001	<0.001

# Indicates that one broiler was excluded from the experiment due to locomotory issues.

1 6CC and 6OS = 6 weeks housing of the broilers in a CC barn or OS barn, respectively. 3OS, 2OS and 1 OS = broilers that were housed in the OS barn for 3, 2 or 1 weeks, respectively, and for the previous 3, 4 or 5 weeks in the CC barn, respectively. REC1 = 4 weeks housing of the broilers in the CC barn, followed by 1 week housing in the OS barn, followed by 1 week in the CC barn. REC2 = 3 weeks housing of the broilers in the CC barn, followed by 2 weeks housing in the OS barn, followed by 1 weeks in the CC barn.

2 Weekly mortality was analyzed independently for each timepoint using a binomial Generalized Linear Model (GLM), with the replicate pen serving as the experimental unit. ^3^*Adj, P value*: weekly *P*-values were adjusted using the Benjamini-Hochberg False Discovery Rate (FDR).

**Fig. 2:**
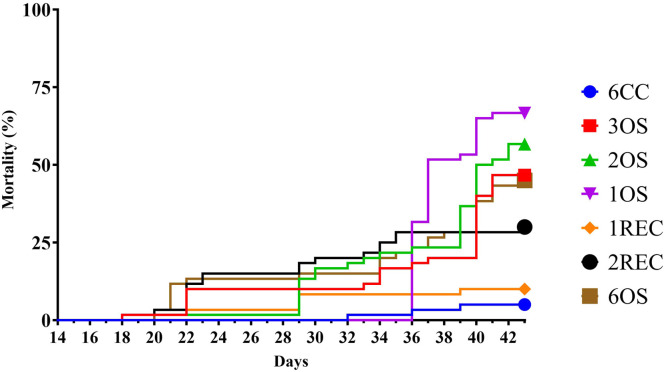
Cumulative mortality recorded in the different treatments during the experimental period. There was no mortality during days 1-14 and therefore these days are not shown. Broilers removed for sampling were not counted as mortality.

Eight broilers were excluded from the experiment due to locomotory issues, induced by rotated tibia or arthritis. The excluded broilers are marked in Table 5 with a hashtag (#) symbol.

### Corticosterone concentrations

Plasma corticosterone concentrations were similar (*P* ≥ 0.389) between the CC and OS treatments of broilers that were housed in the same barn from day 35 to 36 of age (6CC, 2OS and 6OS) (Table 6). Broilers that were transferred to either high temperature (1OS) or thermoneutral temperature (REC2) had higher and lower corticosterone concentrations, respectively. Specifically, broilers of the 1OS treatment (switched from CC to OS) had an increase from 8.23 nmol/L to 25.42 nmol/L (*P* < 0.040), whilst broilers of the REC2 treatment (switched from OS to CC) had a lower concentration, 36.80 nmol/L to 11.56 nmol/L (*P* < 0.003).

**Table 6:** Plasma corticosterone concentration (nmol/L) in broilers either housed in a climate-controlled (CC) barn or an open-sided barn (OS).

	Selected experimental treatments^1^					
	6CC	6OS	2OS	1OS	REC2	SEM^2^
Day						
35	3.44^b^	34.9^a^	19.84^ab^	8.23^by^	36.80^ax^	6.767
36	5.08	30.36	12.41	25.42^x^	11.56^y^	4.701
SEM^3^	0.814	2.290	3.714	8.591	12.620	
***P*-values**						
Treatment	<0.001					
Day	0.331					
Treatment × Day	0.013					

a-b Means within each row without common superscript are significantly different (*P* < 0.05).

x-y Means within each column without common superscript are significantly different (*P* < 0.05).

1 6CC and 6OS = 6 weeks housing of the broilers in the CC barn or OS barn, respectively. 2OS and 1 OS = broilers that were housed in the OS barn for 2 or 1 weeks, respectively, and for the previous 4 or 5 weeks in the CC barn, respectively. REC2 = 3 weeks housing of the broilers in the CC barn, followed by 2 weeks housing in the OS barn, followed by 1 week in the CC barn.

2 The SEM values refer to the pooled standard errors, within day, across treatments.

3 The SEM values refer to the pooled standard errors, within treatment, across days.

Broilers of the 6CC treatment had a low concentration of plasma corticosterone (3.44 nmol/L) on day 35 when compared to the broilers of 6OS and REC2 (34.94 and 36.80 nmol/L, respectively) (*P <* 0.032) (Table 6). The corticosterone concentration of the 2OS and 1OS was in between the concentrations of the aforementioned treatments (19.84 and 8.23 nmol/L).

The following day, day 36, plasma corticosterone concentrations were found to be similar between treatments (*P* ≥ 0.074).

### Protein carbonyl contents

No difference in protein carbonyl contents in blood plasma was observed between day 35 and 36 of age in treatments that remained in the same housing conditions on these ages, i.e., 6CC, 6OS and 2OS (*P* ≥ 0.127) (Table 7). The switch to the OS barn caused an increase in protein carbonyl concentration in the 1OS treatment, from 1.10 to 1.92 nmol/mg protein (*P* < 0.013). The switch from OS to CC (REC2) did not alter the protein carbonyl concentrations on 35 and 36 days (*P* ≥ 0.984).

**Table 7:** Plasma protein carbonyl products (nmol/mg total protein) in broilers either housed in a climate-controlled (CC) barn or an open-sided barn (OS).

	Selected experimental treatments^1^					
	6CC	6OS	2OS	1OS	REC2	SEM^2^
Day						
35	1.08^b^	2.12^a^	1.78^ab^	1.10^bx^	1.15^b^	0.213
36	1.07^b^	1.53^ab^	1.61^ab^	1.92^ay^	1.16^ab^	0.155
SEM^3^	0.007	0.295	0.081	0.401	0.003	
***P*-values**						
Treatment	0.003					
Day	0.937					
Treatment × Day	0.076					

a-b Means within each row without common superscript are significantly different (*P* < 0.05).

x-y Means within each column without common superscript are significantly different (*P* < 0.05).

1 6CC and 6OS = 6 weeks housing of the broilers in the CC barn or OS barn, respectively. 2OS and 1 OS = broilers that were housed in the OS barn for 2 or 1 weeks, respectively, and for the previous 4 or 5 weeks in the CC barn, respectively. REC2 = 3 weeks housing of the broilers in the CC barn, followed by 2 weeks housing in the OS barn, followed by 1 week in the CC barn.

2 The SEM values refer to the pooled standard errors, within day, across treatments.

3 The SEM values refer to the pooled standard errors, within treatment, across days.

### Apparent ileal digestibility

The AID of crude protein was 5.7% higher in REC2 broilers when compared to broilers of the 6OS treatment, which had the lowest AID for crude protein (*P* < 0.040) (Table 8). 6CC and 3OS broilers had intermediate AID of crude protein. The AID of dry matter, ash and organic matter did not differ among the treatments (*P* ≥ 0.283).

**Table 8:** Apparent ileal digestibility^1^ during week six in broilers either housed in a climate-controlled (CC) barn or an open-sided barn (OS). All values are expressed as % of intake.

	Selected experimental treatments^2^				SEM	*P*-values
	6CC	6OS	3OS	REC2		
Dry matter	82.1	79.5	82.2	82.5	1.27	0.343
Ash	74.2	73.0	75.4	74.3	2.37	0.918
Organic matter	82.7	80.1	82.7	83.2	1.22	0.283
Crude protein	88.8^ab^	87.1^b^	91.3^ab^	92.1^a^	1.14	0.040

a-b Means within each row without common superscript are significantly different (*P* < 0.05).

1 The dry matter, ash, organic matter, crude protein and Ti contents of the finisher 2 diet were (g/kg as fed); 926.1, 67.7, 858.4, 186.6 and 1.75, respectively.

2 6CC and 6OS = 6 weeks housing of the broilers in the CC barn or OS barn, respectively. 3OS = broilers that were housed in the OS barn for 3weeks, respectively, and for the previous 3 weeks in the CC barn, respectively. REC2 = 3 weeks housing of the broilers in the CC barn, followed by 2 weeks housing in the OS barn, followed by 1 week in the CC barn.

### Histological examination

Due to the lack of tissue section standardization and the lack of overall variation in results between the treatments the results for both lung and heart are presented in a descriptive manner.

Heart sections mainly represented the left ventricle and parts of the right ventricle. Extravascular subendocardial accumulations of erythrocytes (hemorrhages), spanning several tens of micrometers, were observed in all four surviving broilers of the 6OS treatment and in three out of five broilers of the 2OS treatment; these accumulations were not observed in any of the six 6CC broilers (Fig. 3). These erythrocytes appeared normal with no signs of degeneration and no associated inflammatory cell infiltration was observed in any of the broilers. In the myocardium of six broilers, small areas of extramedullary hematopoietic tissue were present. In addition, hypereosinophilia was observed in the myocardial cells of several heart sections, but the quantitative variation of both presence of hypereosinophilic cardiomyocytes and presence of extramedullary hematopoietic cells was greater within groups than between groups.

**Fig. 3:**
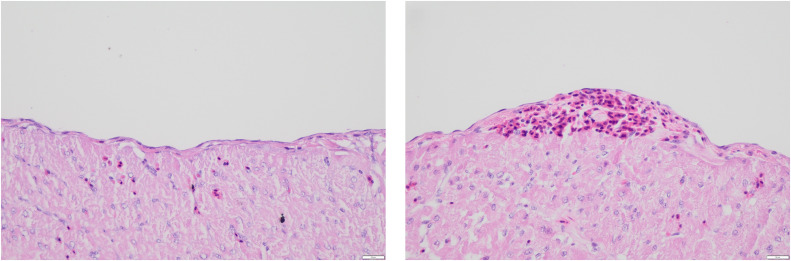
Representative histological pictures of the hearts of the broilers, stained with Haematoxylin and Eosin, 400x magnification (scale bar = 20 µm). Subendocardial myocardium of broilers housed in CC conditions (6CC) (left panel) and housed under HS conditions (6OS) (right panel). The subendocardial myocardium of the bird housed under HS conditions presents with an accumulation of extravasated erythrocytes (haemorrhage) without associated inflammatory cell infiltration or cardiomyocytes. No accumulations were found in any of the broilers housed in CC conditions.

Most histological changes in the lungs were consistent across all groups. Mild inflammation was observed in the lungs of all broilers, regardless of treatment group. These changes primarily consisted of focal mononuclear infiltrations or follicular lymphocyte aggregates, located in areas where bronchial-associated lymphoid tissue (BALT) is typically expected. Isolated parabronchi containing small numbers of heterophilic granulocytes were noted in a few birds, and extramedullary hematopoietic tissue was occasionally identified in the lungs and, in two instances, concurrently within the myocardium.

Furthermore, nodules of partially ossifying cartilage and bone matrix that were identified within the interstitium of the air capillaries. Although these structures were present in every group, the frequency of these nodules appeared to follow a treatment-dependent pattern, with the 6CC group exhibiting a numerically higher average count compared to the 2OS and 6OS groups. No exact quantifications could be extracted due to the lack of section standardization and relative small sample size.

## Discussion

The present study provided novel insights into how the timing and duration of cyclic HS exposure differentially influence broiler performance, physiology, histology and recovery capacity. While previous research has primarily focused on the general effects of HS, our results demonstrate that the timing of HS exposure are critical determinants of both growth suppression and recovery. Specifically, this study uniquely integrates performance data with endocrine, oxidative, intestinal responses and histopathological changes, revealing that HS increases in corticosterone and protein carbonyls coincide with impaired growth, yet do not preclude a rapid restoration of growth rate once birds return to thermoneutral conditions. Importantly, the absence of compensatory growth indicates that early or prolonged HS imposes lasting production losses. These findings advance current understanding by showing that recovery from HS is not solely dictated by the severity of stress, but by the interaction between exposure age and duration, thereby highlighting the need to consider heat stress timing in addition to HS intensity when developing mitigation strategies in broiler production.

The combination of the high ambient temperature with the relative humidity, increased cloacal temperature and the increase of plasma corticosterone levels indicate that the broilers experienced HS, when housed in OS. Thus, subjecting broilers to the prevailing climatological conditions of Thailand during March and April is considered to be a suitable experimental setup to investigate the impact of natural cyclic HS on broilers. Results obtained from a natural cyclic HS model have more practical relevance than those of constant HS, as highlighted by de Souza et al. (2016). Who described that, amongst others, energy and nitrogen efficiencies were reduced only in a constant HS model but not in a cyclic model.

The mortality patterns in this study highlight a critical effect of timing and duration of HS exposure. In this study, the different exposure moments corresponded with simulations of real-life heat waves. Exposing broilers to HS at different ages throughout the growth period provided novel insights on how age and therefore bodyweight, has critical impact on the survival of broilers. Exposure to HS at a younger age resulted in a gradual accumulation of mortality whilst the sudden introduction to HS at 35 days of age was detrimental. The detrimental effect on market age broilers was entirely unexpected. The humane endpoints in place, adapted from Beckford et al. (2020), did not effectively predict mortality in all cases, as some broilers did not show any signs of imminent death. Especially during the last transfer from the CC to the OS barn, some broilers were seen alert and panting at the beginning of inspection and were deceased a few minutes later. Moreover, the majority of the broilers (∼80%) died within 24 h after the transfer from CC to OS and roughly half of those birds died between late afternoon (i.e., 21:00) and early morning (i.e., 7:00) and the high mortality rate specifically during these relatively cool hours was unexpected. This observation is not easy to explain but we can speculate that abrupt HS exposure induces deleterious effects that can be detrimental, hours after first exposure, similar to heat stroke in humans (Mahri and Bouchama, 2018).

Consequently, as broilers do not exhibit clear signs of imminent death when exposed to a severe heat challenge at slaughter age, i.e., 6 weeks, it is recommended that future studies avoid using the complete growth period and adhere to a 5 week growth period, given that broilers tend to grow faster in experimental than commercial settings.

The magnitude of the increased mortality in specific groups can likely be attributed to the lack of prior acclimatisation (Abdelqader and Al-Fataftah, 2014) and to a higher body weight (Gogoi et al., 2021). Both factors are known to increase mortality in broilers during HS. This biological susceptibility is further evidenced by the physiological stress response: while chronically HS exposed broilers maintained elevated corticosterone levels, the acutely exposed HS broilers exhibited a rapid, surge in plasma corticosterone immediately following exposure. From a welfare perspective, this acute physiological stress response, coupled with excessive mortality, demonstrates that sudden heat exposure at slaughter age poses an immediate and unmanageable threat to broiler well-being.

In view of our research objectives, a straightforward factorial design was not feasible, as we examined exposure to HS during multiple, overlapping time intervals and durations of both biological interest and practical relevance. Consequently, it was impossible to entirely decouple the treatment from a potential barn-by-treatment interaction. As a result, the environmental treatment is partly linked to barn conditions. Although pens were used as experimental units, they shared the same overall environment, meaning barn-specific effects cannot be fully separated from the treatment. Furthermore, the study design reflects commercial broilers, where factors such as acclimation, barn movement, and age are inherently connected. Therefore, the results should be interpreted as a combined response to these interacting variables. Finally, some procedures, like handling, cloacal temperature measurements, and blood sampling, may have introduced additional stress, although these were standardized across treatments or limited to individual birds.

To the best of the authors’ knowledge, this is the first study that assesses growth performance of broilers during specific periods of HS within the growth period in one experimental setting. In accordance with general consensus, growth performance was negatively affected by HS independent of duration. However, the dynamics of the growth retardation differed greatly depending on the age of exposure. Our data indicate that HS suppresses the relative growth similarly when broilers are exposed to HS at a younger age, irrespective of previous weekly HS exposure. This is not the case when broilers are exposed to HS from hatch, as these broilers have a higher growth ratio than the broilers exposed to HS later in life. This finding is in line with previous studies that underscore the importance of early thermal manipulation (Yahav, 1999; Ouchi et al., 2020).

According to our data, broilers in the recovery treatments did not exhibit compensatory growth within the experimental period. Although their growth ratios normalized immediately to control levels, indicating that the growth potential was not permanently lost, the accumulated body weight deficit was not regained. The fact that the growth ratios returned to control levels, indicates that broilers have a “maximum” growth rate, which cannot be exceeded to compensate for prior losses within a standard broiler production cycle. Jinxue et al. (2023) demonstrated that a 48-hour period of thermoneutrality was insufficient for broilers to recover from the growth inhibition caused by 72 hours of heat stress exposure. These results complement our finding that a longer period, i.e., one-week of recovery, is still inadequate to restore the body weight deficit.

Regarding the histological changes, we expected that due to the excessive strain put on the cardiorespiratory system more distinct histological changes would be present between the experimental treatments. However, only the histological examination of the hearts revealed a morphologic feature that seemed exclusively present in broilers exposed to high ambient temperatures for either 1 or 5 weeks: mild multifocal subendocardial hemorrhages not accompanied by myocyte degeneration or inflammatory cell infiltrates. Across species, including humans, subendocardial hemorrhages are associated with several conditions, among which shock and intravascular coagulation are two prominent potential causes (Cioffi et al., 2024). In the current study these subendocardial hemorrhages might therefore be explained as associated with heat shock. However, in order to solidify and further quantify these findings, additional research with more broilers is necessary. Unfortunately, similar to findings in human forensic medicine, death after a period of HS does not present with pathognomonic cardiac lesions. Therefore, the diagnosis of HS-associated heart damage must rely on the clinicopathological context (Marchand and Gin, 2022).

Regarding the ossifying cartilage and bone nodules, these can be incidentally found in lungs of broilers (Maxwell et al., 1989). Although the variations noted between groups in the current study suggests a treatment-related effect, a sound relationship between these nodules and the thermal treatments could not be established.

Protein carbonyl products were measured in our study as an indicator of oxidative stress (Dalle-Donne et al., 2003). Our data demonstrate that plasma protein carbonyl products increase not only after a prolonged heat challenge but also following an acute heat challenge. This aligns with the findings of Cartonelli Mancinelli et al. (2023) and underscores the utility of protein carbonyl products as a reliable biomarker for HS in plasma.

Finally, regarding nutrient digestibility, our results largely corroborate the findings of de Souza et al. (2016), who reported that cyclic HS does not compromise the AID of dry matter, ash, or organic matter. Furthermore, AID for these nutrients was comparable between broilers returned to CC conditions and those maintained under chronic HS. This suggests that digestive capacity is not permanently impaired by the thermal challenge. However, broilers that returned to CC conditions showed a higher CP digestibility compared to the chronically heat stressed broilers, which indicates that heat load may specifically impair protein digestion or absorption. Typically, a reduction in feed intake is associated with a slower passage rate and, consequently, increased nutrient digestibility (Mens et al., 2022). However, our data suggest that HS negatively affects digestibility independently of feed intake. Taken together, impaired nutrient digestibility is not the primary cause of the growth retardation observed during cyclic HS.

In conclusion, the objective of the study was to evaluate the effects of timing and duration of cyclic HS on growth performance and recovery. Our study demonstrates that the timing and duration of cyclic HS exposure are critical determinants of broiler survival and growth. Although corticosterone and protein carbonyl was increased due to HS, after a period of HS broilers can exhibit rapid recovery of growth rate. Nevertheless, compensatory growth was not observed. Cyclic HS did not cause pathognomonic cardiopulmonary lesions, though mild subendocardial hemorrhages indicative of heat shock were exclusively observed in heat-stressed broilers.

## CRediT authorship contribution statement

**A. Papanikolaou:** Writing – original draft, Visualization, Methodology, Formal analysis, Data curation, Conceptualization. **E.M.J.M. Kampschoer:** Writing – original draft, Visualization, Methodology, Formal analysis, Data curation, Conceptualization. **M.G.R. Matthijs:** Writing – review & editing, Supervision, Project administration, Methodology, Funding acquisition, Conceptualization. **S. Khempaka:** Writing – review & editing, Supervision, Project administration. **E.A.W.S. Weerts:** Writing – review & editing, Methodology, Formal analysis, Data curation. **J.T. Schonewille:** Writing – review & editing, Supervision, Project administration, Methodology, Funding acquisition, Conceptualization.

## Disclosures

The authors declare that they have no conflicts of interest to report.

## Appendix Supplementary materials

Image, image 1 dummy alt text

Image, application 2
