# Supplementary figures and images for: Impact of duration of cyclic heat stress exposure at different ages on growth performance, recovery, and histopathology in broilers
Source: Poult Sci. 2026 Jul 2;105(10):107367. doi: 10.1016/j.psj.2026.107367 (PMC13356763; doi:10.1016/j.psj.2026.107367)

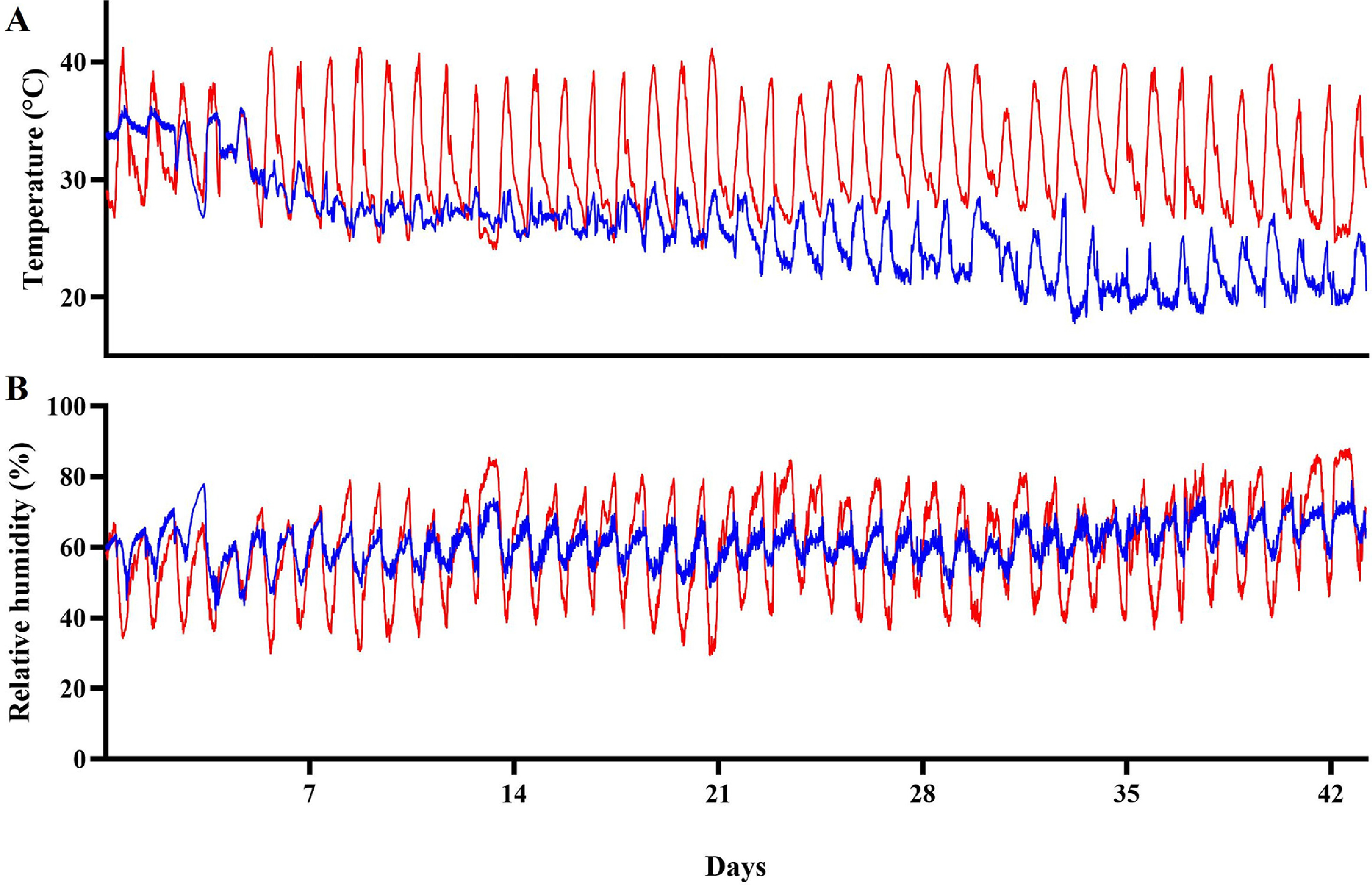

Supplement: Supplementary file 1 [file mmc1.jpg]
