# Supplementary material for: Impact of duration of cyclic heat stress exposure at different ages on growth performance, recovery, and histopathology in broilers
Source: Poult Sci. 2026 Jul 2;105(10):107367. doi: 10.1016/j.psj.2026.107367 (PMC13356763; doi:10.1016/j.psj.2026.107367)
Supplement: Supplementary file 2 [file mmc2.docx]

Supplementary Table 1. Cloaca temperature (°C), measured at 8:00 hr and 16:00 hr, in broilers either housed in a climate controlled (CC) barn or an open sided barn (OS)

|  | Experimental treatments^1^ | | | | | | |
| --- | --- | --- | --- | --- | --- | --- | --- |
|  | 6CC | 6OS | 3OS | 2OS | 1OS | REC1 | REC2 |
| Day (hr) |  |  |  |  |  |  |  |
|  |  |  |  |  |  |  |  |
| 21 (16.00) | 41.3 | 43.8 | 40.8 | 40.9 | 41.0 | 41.2 | 41.1 |
| 22 (8.00) | 40.7 | 40.8 | 40.9 | 41.0 | 41.1 | 41.1 | 40.8 |
| 22 (16.00) | 41.4 | 42.9 | 42.8 | 41.6 | 41.3 | 41.5 | 43.1 |
|  |  |  |  |  |  |  |  |
| 28 (16.00) | 41.1 | 42.7 | 42.5 | 40.9 | 40.9 | 41.1 | 42.7 |
| 29 (8.00) | 41.0 | 41.2 | 41.5 | 40.7 | 41.1 | 41.1 | 41.7 |
| 29 (16.00) | 41.1 | 43.4 | 43.3 | 43.1 | 41.1 | 43.4 | 43.4 |
|  |  |  |  |  |  |  |  |
| 35 (16.00) | 41.2 | 42.6 | 43.6 | 43.2 | 41.0 | 42.9 | 43.4 |
| 36 (8.00) | 41.0 | 41.6 | 41.8 | 41.7 | 41.1 | 41.8 | 42.2 |
| 36 (16.00) | 41.5 | 43.9 | 43.3 | 43.1 | 43.4 | 40.5 | 40.3 |
|  |  |  |  |  |  |  |  |
| 42 (16.00) | 41.4 | 43.1 | 43.1 | 43.3 | 43.1 | 41.1 | 41.1 |
| 43 (8.00) | 40.9 | 41.5 | 41.6 | 41.7 | 42.1 | 41.2 | 41.1 |
| 43 (16.00) | 41.6 | 43.4 | 43.0 | 43.1 | 43.1 | 41.5 | 41.7 |

^1^ 6CC and 6OS = 6 weeks housing of the broilers in the CC barn or OS barn, respectively. 3OS, 2OS and 1 OS = broilers that were housed in the OS barn for 3, 2 or 1 weeks, respectively, and for the previous 3, 4 or 5 weeks in the CC barn, respectively. REC1 = 4 weeks housing of the broilers in the CC barn, followed by 1 week housing in the OS barn, followed by 1 week in the CC barn. REC2 = 3 weeks housing of the broilers in the CC barn, followed by 2 weeks housing in the OS barn, followed by 1 weeks in the CC barn.
